# Update on Vaccine-Derived Polioviruses — Worldwide, January 2016–June 2017

**DOI:** 10.15585/mmwr.mm6643a6

**Published:** 2017-11-03

**Authors:** Jaume Jorba, Ousmane M. Diop, Jane Iber, Elizabeth Henderson, Roland W. Sutter, Steven G.F. Wassilak, Cara C. Burns

**Affiliations:** ^1^Division of Viral Diseases, National Center for Immunization and Respiratory Diseases, CDC; ^2^Department of Polio Eradication, Detection and Interruption Unit, World Health Organization (WHO), Geneva, Switzerland; ^3^Department of Polio Eradication, Research, Policy and Containment Unit, WHO, Geneva, Switzerland; ^4^Global Immunization Division, Center for Global Health, CDC.

In 1988, the World Health Assembly launched the Global Polio Eradication Initiative (GPEI) (*1*). Among the three wild poliovirus (WPV) serotypes, only type 1 (WPV1) has been detected since 2012. Since 2014, detection of WPV1 has been limited to three countries, with 37 cases in 2016 and 11 cases in 2017 as of September 27. The >99.99% decline worldwide in polio cases since the launch of the GPEI is attributable to the extensive use of the live, attenuated oral poliovirus vaccine (OPV) in mass vaccination campaigns and comprehensive national routine immunization programs. Despite its well-established safety record, OPV use can be associated with rare emergence of genetically divergent vaccine-derived polioviruses (VDPVs) whose genetic drift from the parental OPV strains indicates prolonged replication or circulation (*2*). VDPVs can also emerge among persons with primary immunodeficiencies (PIDs). Immunodeficiency-associated VDPVs (iVDPVs) can replicate for years in some persons with PIDs. In addition, circulating vaccine-derived polioviruses (cVDPVs) can emerge very rarely among immunologically normal vaccine recipients and their contacts in areas with inadequate OPV coverage and can cause outbreaks of paralytic polio. This report updates previous summaries regarding VDPVs (*3*). During January 2016–June 2017, new cVDPV outbreaks were identified, including two in the Democratic Republic of the Congo (DRC) (eight cases), and another in Syria (35 cases), whereas the circulation of cVDPV type 2 (cVDPV2) in Nigeria resulted in cVDPV2 detection linked to a previous emergence. The last confirmed case from the 2015–2016 cVDPV type 1 (cVDPV1) outbreak in Laos occurred in January 2016. Fourteen newly identified persons in 10 countries were found to excrete iVDPVs, and three previously reported patients in the United Kingdom and Iran (*3*) were still excreting type 2 iVDPV (iVDPV2) during the reporting period. Ambiguous VDPVs (aVDPVs), isolates that cannot be classified definitively, were found among immunocompetent persons and environmental samples in 10 countries. Cessation of all OPV use after certification of polio eradication will eliminate the risk for new VDPV infections.

WPV type 2 (WPV2) was last detected in 1999 and global WPV2 eradication was declared in September 2015; WPV type 3 has not been detected since 2012. Since August 2014, residual endemic WPV1 transmission has been detected only in Afghanistan, Pakistan, and Nigeria, mostly in inaccessible areas. In response to the emergence of multiple cVDPV2 outbreaks, the World Health Organization (WHO) coordinated the synchronized withdrawal of the type 2 component (OPV2; Sabin type 2) from trivalent OPV (tOPV; Sabin types 1, 2, and 3) (*4*). In April 2016, all OPV-using countries switched to bivalent OPV (bOPV; Sabin types 1 and 3). Since the switch, the number of isolated Sabin 2 strains from both acute flaccid paralysis and environmental surveillance systems has steadily declined (*5*). To monitor the disappearance of Sabin 2 strains and to ensure identification of type 2 VDPVs (VDPV2s), as of August 1, 2016, all poliovirus type 2 (PV2) isolates are referred for genetic sequencing.

## Properties and Virologic Characterization of VDPVs

Poliovirus isolates are grouped into three categories: WPV, vaccine-related poliovirus (VRPV), and VDPV. VRPVs have limited divergence in the capsid protein (VP1) nucleotide sequences from the corresponding OPV strain (poliovirus type 1 and 3 [PV1 and PV3]: ≤1% divergent; poliovirus type 2: ≤0.6% divergent) (*3*). VDPVs are >1% divergent (for PV1 and PV3) or >0.6% divergent (for PV2) in VP1 nucleotide sequences from the corresponding OPV strain (*3*)*.* VDPVs are further classified as 1) cVDPVs, when evidence of person-to-person transmission in the community exists; 2) iVDPVs, when they are isolated from persons with PIDs; and 3) aVDPVs, when they are clinical isolates from persons with no known immunodeficiency and no evidence of transmission, or they are sewage isolates that are unrelated to other known VDPVs and whose source is unknown (*2*). GPEI guidelines about reporting and classification of VDPVs were last updated in August 2016 (http://polioeradication.org/wp-content/uploads/2016/09/Reporting-and-Classification-of-VDPVs_Aug2016_EN.pdf).

All poliovirus isolates are characterized by laboratories of the Global Polio Laboratory Network. VDPV screening is conducted using real-time reverse transcription–polymerase chain reaction (RT-PCR) nucleic acid amplification, targeted to nucleotide substitutions that frequently revert to the parental WPV sequence during replication of OPV in the human intestine (*6*). Starting August 1, 2016, the use of the VDPV2 screening assay was discontinued and all PV2 isolates are sequenced. Potential VDPVs identified by real-time RT-PCR screening are sequenced in the VP1 region for definitive analysis.

## Detection of cVDPVs

**FIGURE 1:**
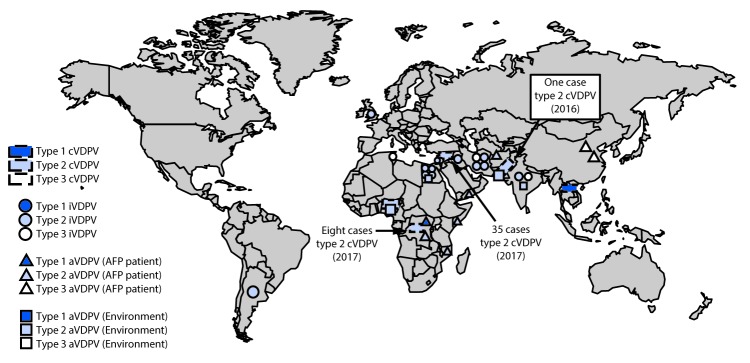
Vaccine-derived polioviruses (VDPVs) detected, by serotype and VDPV classification* — worldwide, January 2016–June 2017 **Abbreviations:** AFP = Acute flaccid paralysis; aVDPV = ambiguous VDPV; cVDPV = circulating VDPV; iVDPV = immunodeficiency-associated VDPV. * Spread of cVDPVs followed the elimination of the corresponding serotype of indigenous wild poliovirus, but with continued introduction of oral poliovirus vaccine into communities with growing immunity gaps. All of the cVDPV outbreaks were detected first by the laboratory, using sequence data and evolutionary analyses.

**FIGURE 2:**
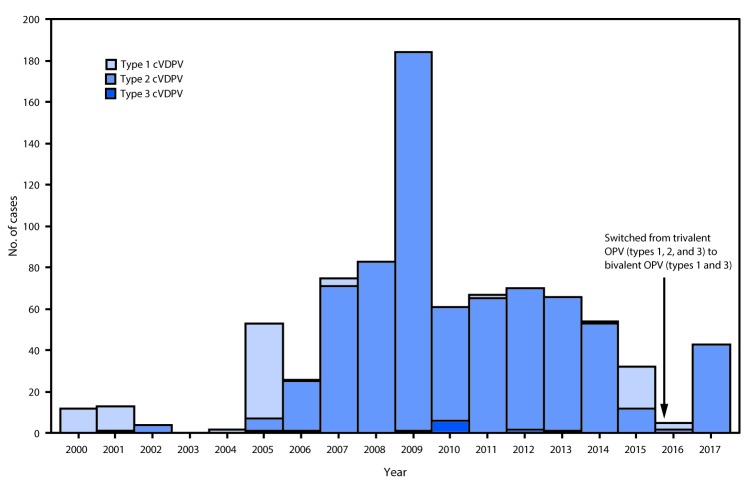
Circulating vaccine-derived poliovirus (cVDPV) cases detected, by serotype — worldwide, January 2000–June 2017*,^†^ **Abbreviation:** OPV= oral poliovirus vaccine. * Data available by August 25, 2017. ^^†^^ In April 2016, all OPV-using countries switched from trivalent OPV (types 1, 2, and 3) to bivalent OPV (types 1 and 3).

During January 2016–June 2017, the number of countries with detected cVDPV circulation decreased from seven to five since the previous reporting period (*3*) (Figure 1); all except one (cVDPV1 in Laos) reported cVDPV2 circulation (Table). No additional cases have been identified from previously reported VDPV outbreaks in Guinea (cVDPV2), Madagascar (cVDPV1), Myanmar (cVDPV2), Ukraine (cVDPV1), Pakistan (cVDPV2) and Nigeria (cVDPV2). Cases continued to be identified from the previously reported distinct cVDPV2 outbreak in Nigeria (*7*) and the previously reported cVDPV1 outbreak in Laos (*3*). New outbreaks were reported in DRC (two cVDPV2 emergences; one with six cases and one with two cases), Nigeria (cVDPV2, one case), Pakistan (cVDPV2, one case), and Syria (cVDPV2, 35 cases) (Table). Detection of the new cVDPV2 outbreaks occurred after the global tOPV to bOPV switch (April 2016). During January 2016–June 2017, among 48 cVDPV cases, 45 (93%) were cVDPV2 (Table) (Figure 2). Selected cVDPVs from the reporting period are described below.

**TABLE:** Vaccine-derived polioviruses (VDPVs) detected, by classification and other selected characteristics — worldwide, January 2016–June 2017

Category	Country	Year(s) detected*	Source^†^		Serotype	No. of isolates^§^ January 2016–June 2017			Capsid protein VP1 divergence from Sabin OPV strain (%)	Coverage with 3 doses of OPV (%)**	Estimated duration of VDPV replication^††^ (yrs)	Current status (date of last outbreak case, patient isolate, or environmental sample)
						No. of cases	No. of contacts	Non-AFP source				
**cVDPV**	Democratic Republic of the Congo	2017	Outbreak		2	6	0	0	2.1	74	1.9	06/26/17
	Democratic Republic of the Congo	2017	Outbreak		2	2	1	0	0.7	74	0.6	04/18/17
	Laos	2015–16	Outbreak		1	3	4	0	2.3–3.9	83	3.5	02/06/16
	Nigeria	2016	Outbreak		2	1	1	0	1.3–1.8	49	1.6	11/24/16
	Nigeria	2013–16	Outbreak– importation		2	0	1	1	3.5–4.1	49	3.7	08/26/16
	Pakistan	2016	Outbreak		2	1	0	4	1.0–2.0	72	1.8	12/28/16
	Syria	2017	Outbreak		2	35	27	0	2.3–3.1	48	2.8	06/30/17
**Total cVDPV**	**—^§§^**	**—^§§^**	**—^§§^**		**—^§§^**	**48**	**34**	**5**	**—^§§^**	**—^§§^**	**—^§§^**	**—^§§^**
**iVDPV**	Argentina	2016	Non-AFP AGG		2	1	0	0	0.9	87	0.8	10/22/16
	Egypt	2016	Non-AFP SCID		2	0	0	1	2.0	95	1.8	05/21/16
	Egypt	2016	Non-AFP SCID		2	0	0	1	0.6	95	0.5	07/17/16
	Egypt	2017	AFP patient		2	1	0	0	1.9	95	1.7	02/13/17
	India	2016	AFP patient XLA		2	1	0	0	0.7	86	0.6	03/08/16
	India	2015–2016	Non-AFP SCID		3	0	0	1	4.5–10.2	86	9	08/04/16
	Iran	2016	AFP patient		2	1	0	0	0.6	99	0.5	11/26/16
	Iran	2015–2016	Non-AFP PID		2	0	0	1	1.5	99	1.4	02/18/16
	Iran	2015–2017	Non-AFP PID		2	0	0	1	2.5	99	2.3	02/12/17
	Iran	2015–2016	Non-AFP PID		3	0	0	1	2.6	99	2.4	08/07/16
	Iraq	2016	AFP patient		2	1	0	0	0.7	68	0.6	02/02/16
	Israel	2017	Non-AFP PID		2	0	0	1	2.4	94	2.2	01/23/17
	Nigeria	2016	AFP patient		2	1	0	0	0.9	49	0.8	05/14/16
	Pakistan	2016	AFP patient		2	1	0	0	1.1	72	1	09/07/16
	Tunisia	2016–2017	AFP patient XLA		3	1	0	0	1.2	98	1.1	01/11/17
	United Kingdom	2015–2017	Non-AFP PID		2	0	0	1	17.94	94	>30	05/11/17
	West Bank and Gaza Strip	2016–2017	Non-AFP SCID		2	0	0	1	1.0	94	0.9	02/08/17
**Total iVDPV**	**—^§§^**	**—^§§^**	**—^§§^**		**—^§§^**	**8**	**0**	**9**	—**^§§^**	**—^§§^**	**—^§§^**	**—^§§^**
**aVDPV**	Afghanistan	2016	AFP patient		2	1	0	0	1.0	60	0.9	09/10/16
	China	2016	AFP patient		3	1	0	0	1.2	99	1	08/16/16
	China	2017	AFP patient		3	1	0	0	1.1	99	1	02/19/16
	Democratic Republic of the Congo	2016	AFP patient		2	2	0	0	0.6–1.7	74	0.5–1.5	03/15/16
	Democratic Republic of the Congo	2017	AFP patient		1	1	0	0	2.7	74	2.5	04/01/17
	Egypt	2016	Environmental sample		2	0	0	1	0.6	95	0.5	03/15/16
	India	2016–2017	Environmental sample		2	0	0	7	0.7–1.5	86	0.6–1.4	03/29/17
	Mozambique	2016	AFP patient		2	1	1	0	1.3	80	1.1	11/30/16
	Nigeria	2017	Non-AFP		2	0	1	0	0.7	49	0.7	03/02/17
	Nigeria	2017	Environmental sample		2	0	0	11	0.6–1.1	49	0.5–1	04/17/17
	Pakistan	2016–2017	Environmental sample		2	0	0	8	0.6–1.3	72	0.5–1.1	05/29/17
	Russian Federation	2016	AFP patient		2	1	1	0	1.1–1.4	97	1–1.2	12/08/16
	Somalia	2016	AFP patient		2	1	0	0	1.1	47	1	10/27/16
	Yemen	2016	AFP patient		2	1^¶¶^	1^¶¶^	0	0.8–0.9	65	0.9	06/20/16
**Total aVDPV**	**—^§§^**	**—^§§^**		**—^§§^**	**—^§§^**	**10**	**4**	**27**	**—^§§^**	**—^§§^**	**—^§§^**	**—^§§^**

**Abbreviations:** AFP = acute flaccid paralysis; AGG = agammaglobulinemia; aVDPV = ambiguous VDPV; cVDPV = circulating VDPV; IPV = inactivated poliovirus vaccine; iVDPV = immunodeficiency-associated VDPV; OPV = oral poliovirus vaccine; PID = primary immunodeficiency; SCID = severe combined immunodeficiency; XLA = X-linked agammaglobulinemia.

* Total years detected for previously reported cVDPV outbreaks (Nigeria).

^†^ Outbreaks list total cases clearly associated with cVDPVs. Some VDPV case isolates from outbreak periods might be listed as aVDPVs.

^§^ Total cases for VDPV-positive specimens from AFP cases and total VDPV-positive samples for environmental (sewage) samples.

^¶^ Percentage of divergence is estimated from the number of nucleotide differences in the VP1 region from the corresponding parental OPV strain.

** Coverage with 3 doses of OPV, based on 2016 data from the World Health Organization (WHO) Vaccine Preventable Diseases Monitoring System (2016 global summary) and WHO-United Nations Children’s Fund coverage estimates, http://www.who.int/gho/immunization/poliomyelitis/en/. National data might not reflect weaknesses at subnational levels.

^††^ Duration of cVDPV circulation was estimated from extent of VP1 nucleotide divergence from the corresponding Sabin OPV strain; duration of iVDPV replication was estimated from clinical record by assuming that exposure was from initial receipt of OPV; duration of aVDPV replication was estimated from sequence data.

^§§^ Not cumulative data.

^¶¶^ Two genetically linked isolates were classified as aVDPVs according to the VDPV guidelines (http://polioeradication.org/wp-content/uploads/2016/09/Reporting-and-Classification-of-VDPVs_Aug2016_EN.pdf), which require detection for >2 months.

**Democratic Republic of the Congo.** Circulating VDPV2s were isolated from eight acute flaccid paralysis (AFP) patients and one contact during February–June 2017. cVDPV2s represented two distinct emergences (0.7%–2.1% VP1 nucleotide divergence from parental Sabin 2 strain): one circulating in Haut Lomami province (six cases; latest case onset June 26, 2017)* and one circulating in Maniema province (isolated from two patients and one contact; latest case onset April 18, 2017). Reported OPV coverage was low (74%); two monovalent OPV type 2 (mOPV2) mass vaccination campaigns were conducted during July 13–29, 2017 and mop-up vaccination campaigns were conducted during September 17–20, 2017.

**Nigeria.** During the reporting period, cVDPV2s (with 3.5%–4.1% VP1 nucleotide divergence from a cVDPV2 emergence originating in Chad in 2012) were found only in the northern state of Borno. The cVDPV2s were isolated in districts of Borno proximal to inaccessible areas, one from an environmental sample collected on April 23, 2016 in Maiduguri, and one from a contact sample collected on August 26, 2016 in Monguno, after detection of a WPV1 case in the same area. An independent cVDPV2 emergence (with 1.3%–1.8% VP1 nucleotide divergence) was reported in Sokoto with virus detected from a patient with onset of AFP October 28, 2016 and a nonhousehold contact sample collected on November 24, 2016. Estimated divergence of the cVDPV2s in Sokoto from Sabin 2 indicate OPV2 origin at least 6 months before the tOPV to bOPV switch in April 2016.

**Pakistan.** During October 2016–December 2016, a new cVDPV2 emergence was reported in Quetta, the provincial capital of Baluchistan. Five cVDPV2s (with 1.0%–2.0% VP1 nucleotide divergence) were detected, four from sewage samples collected in two distinct environmental sites during three consecutive months (most recent sample date December 28, 2016) and one from an AFP patient with paralysis onset on December 17, 2016.

**Syria.** Syria is facing a humanitarian crisis because of armed conflict, and during March 2017–June 2017, cVDPV2s were isolated from 35 AFP patients and 27 contacts in two governorates (Deir ez-Zor and Raqqa).^†^ The outbreak was associated with an emergence first observed in a child aged 22 months with onset of paralysis on March 3, 2017. Among 32 AFP cases, 29 (90%) were identified in the Mayadeen district of Deir Ez-Zor governorate. The extent of VP1 nucleotide divergence from the parental Sabin 2 strain among all cVDPV2s was 2.3%–3.1% VP1 nucleotide divergence. Reported OPV coverage was low (48%) and in response to the outbreak, mOPV2 mass vaccination campaigns were conducted during July (Deir Ez-Zor) and August (Raqqa), reaching an estimated 350,000 children. 

## Detection of iVDPVs

During January 2016–June 2017, 17 iVDPV infections were reported from 11 countries (Table), including 14 that were newly detected iVDPV infections. During this reporting period, with the exception of three type 3 iVDPVs (iVDPV3), all were type 2. Since introduction of OPV, the cumulative serotype distribution shows that iVDPV2 are the most common (69%), followed by type 3 (14%) type 1 (12%) and heterotypic mixtures (i.e., types 1 and 2 or types 2 and 3) (5%). Selected iVDPVs from the reporting period are described below.

**Egypt**. A boy aged 11 months infected with iVDPV2 developed AFP in February 2017. In addition, three patients with PID who did not have AFP were newly identified as infected with iVDPV2s.

**India.** A girl aged 65 months with agammaglobulinemia was infected with iVDPV2 and developed AFP in February 2016. An iVDPV3 infection in a patient with severe combined immunodeficiency without AFP was first detected in January 2015; the last sample from this patient that was positive for iVDPV3 was collected in August 2016. Samples collected since October 2016 were negative for type 3 VDPVs (VDPV3).

**Iran.** A boy aged 14 months with PID, who received his fourth OPV dose in September 2016, and was infected with an iVDPV2, developed AFP in November 2016.

**Iraq.** A girl aged 7 months with PID and infected with iVDPV2 developed AFP in February 2016.

**Pakistan.** An iVDPV2 was isolated from a boy aged 7 months with PID after onset of AFP in February 2016.

**Tunisia.** A girl aged 6 months with PID and infected with iVDPV3 developed AFP in November 2016. The last VDPV-positive specimen was collected in January 2017.

## Detection of aVDPVs

During January 2016–June 2017, aVDPVs were isolated in 11 countries (Table). The most divergent aVDPV (2.7% VP1 divergence) was isolated from an AFP patient in DRC. This represented an emergence independent of cVDPV2 circulating in the country during the same period. Detection of aVDPVs in settings with <60% polio vaccination coverage might indicate a risk for cVDPV emergence and further spread as well as potential gaps in surveillance. Selected aVDPVs from the reporting period are described below.

**Afghanistan.** A type 2 aVDPV (aVDPV2), with 1.0% VP1 divergence, was isolated in a girl aged 30 months who developed AFP in September 2016.

**China.** Two type 3 aVDPVs (aVDPV3s), with 1.1%–1.2% VP1 divergence, were isolated from two AFP patients in Henan and Inner Mongolia provinces with onset dates in February 2017 and August 2016, respectively.

**DRC.** Two aVDPV2s, with 0.6%–1.7% VP1 divergence, were isolated from AFP patients in two different provinces during January–March 2016. An aVDPV1 (with 2.7% VP1 divergence) was isolated in a boy aged 32 months who developed AFP in April 2017.

**India.** Seven aVDPV2s, with 0.7%–1.5% VP1 divergence, were isolated from environmental samples collected in three different cities (four collection sites in Delhi, one in Kolkata, and two in Hyderabad) during the reporting period.

**Nigeria.** Twelve aVDPV2s (11 from sewage samples and one from a contact, and all with 0.6%–1.1% VP1 divergence) were isolated in Bauchi (one), Gombe (two), Katsina (one), and Sokoto (eight) states during the reporting period.

**Pakistan.** Eight aVDPV2s, with 0.6%–1.3 VP1 divergence, were detected in environmental samples collected in Quetta (six), Pishin (one), and Hyderabad (one) during June 2016–May 2017.

**Yemen.** Two aVDPV2s, with 0.8%–0.9% VP1 divergence, were detected, one from an AFP patient with onset date June 11, 2016, and one from a contact sample collected June 20, 2016.

## Discussion

The number of reported cVDPV outbreaks has decreased from nine to seven since the January 2015–May 2016 reporting period (*3*); however, the total number of reported cVDPV cases in these outbreaks has increased. Control and interruption of cVDPV2 outbreaks in areas at high risk for cVDPV emergence is partly attributable to steadily improving quality of supplementary immunization activities^§^ and increased access to unimmunized children. For example, the large cVDPV2 outbreaks reported in Nigeria, Chad, and Pakistan during the last 5 years were interrupted; however, residual detection of cVDPV2s at the subnational level is indicative of persistent pockets of underimmunized children in mostly inaccessible areas (*7*). The new cVDPV2 outbreaks in DRC and Syria highlight the importance of maintaining high levels of poliovirus immunity, as well as sensitive AFP surveillance.

Expanded environmental surveillance in countries at high risk for poliovirus (PV) importation or VDPV emergence has increased the sensitivity of poliovirus detection and has played a critical role in monitoring residual PV2 excretion after OPV2 cessation. Despite the logistical and technical challenges of detecting and sequencing polioviruses from sewage samples, environmental surveillance remains critical in not only increasing the sensitivity of both WPV and VDPV detection, but also accelerating the GPEI response (*8*).

During the reporting period, VDPV2s were detected both before and after the global withdrawal of OPV2 in April 2016. During the preswitch period (January 2016–April 2016), emergence of cVDPV2 in countries with low routine vaccination coverage underscored the risks of widening immunization gaps to PV2; detection of highly divergent cVDPV2s from sewage samples collected in the state capital adjacent to inaccessible areas of northeast Nigeria also indicated virus circulation that was missed by AFP surveillance. cVDPV2 outbreaks detected after the switch in both Syria and DRC highlighted the risks associated with chronically low tOPV vaccination coverage before the switch. Outbreak response to VDPV2 transmission after OPV2 cessation requires use of mOPV2; the scope and intensity of mOPV2 vaccination campaigns in outbreak areas is assessed by the mOPV2 Advisory Group, which advises the WHO Director General on release of mOPV2. Response to the cVDPV2 outbreak in Syria included two mOPV2 mass vaccination campaigns targeting >300,000 children aged <5 years, followed by inactivated polio vaccine (IPV) vaccination campaigns targeting >100,000 children aged 2–24 months, including populations at high risk in adjacent areas and countries.

In April 2016, all 155 OPV-using countries and territories switched from tOPV to bOPV; the number of countries reporting PV2 detection decreased 83%, from 42 before the switch to seven after the switch (January–March 2017) (*5*). The GPEI and Global Polio Laboratory Network have continued to strengthen AFP and poliovirus surveillance. In addition, the increase in the number of environmental surveillance sites has enhanced PV detection (*9*). Routine immunization services also are being strengthened, and most countries incorporated at least 1 dose of IPV into routine childhood immunization schedules in 2016. Augmented surveillance for VDPV infections among patients with PID (*10*) has increased the number of known iVDPV excretors. Continued progress in development of antivirals is needed to eliminate virus shedding in persons with chronic iVDPV infections.

During the last 5 years, the number of WPV cases (>400 in 2013; 12 in 2017) was lower than the estimated number (250–500) of global vaccine-associated paralytic poliomyelitis cases.^¶^ The ultimate goal of the polio endgame strategic plan is the global cessation of all OPV use after the end of all WPV circulation, which started with cessation of OPV with a type 2 component. Cessation of all OPV use after certification of polio eradication will eliminate the risk for cVDPV outbreaks, and new iVDPV and aVDPV infections.

**Summary**

**What is already known about this topic?**

Vaccine-derived polioviruses (VDPVs), strains that are genetically divergent from the oral poliovirus vaccine (OPV) viruses, fall into three categories: 1) circulating VDPVs (cVDPVs) from outbreaks, 2) immunodeficiency-associated VDPVs (iVDPVs) from patients with primary immunodeficiency diseases (PIDs), and 3) ambiguous VDPVs (aVDPVs), which cannot be more definitively identified. cVDPVs are biologically equivalent to wild polioviruses, emerge in settings of low population immunity, and can sustain long-term circulation. Because >94% of cVDPVs since 2006 and 69% of iVDPVs since OPV introduction are type 2, the World Health Organization coordinated worldwide replacement of trivalent OPV (tOPV, types 1, 2, and 3) with bivalent OPV (bOPV, types 1 and 3) in April 2016.

**What is added by this report?**

During 2017, new cVDPV outbreaks were detected in the Democratic Republic of the Congo (two emergences) and Syria (one emergence). Residual circulation of a previous cVDPV2 emergence in Nigeria was detected in 2016 and low-level detection of new emergences in Nigeria and Pakistan occurred during 2016. Fourteen newly identified persons in 10 countries were found to excrete iVDPVs.

**What are the implications for public health practice?**

The goal of the Global Polio Eradication Initiative is the cessation of all poliovirus circulation. The risk for VDPV emergence will continue as long as OPV is used. The switch from tOPV to bOPV in April 2016 was the first step toward phasing out the use of all OPV, setting the stage for a subsequent total worldwide shift from OPV to IPV.

## Footnotes

* As of September 15, one new cVDPV2 was isolated from an AFP case detected in Haut Lomami province with onset date August 9, 2017.

† As of September 15, four new cVDPV2s were isolated from four AFP cases detected in Deir ez-Zor (three cases) and Homs (one case) governorates. The total number of AFP cases is 39. The onset date of the latest AFP case was July 13, 2017.

§ Supplementary immunization activities are mass vaccination campaigns conducted in a short period (days to weeks) during which a dose of OPV is administered to all children aged <5 years, regardless of previous vaccination history. Campaigns can be conducted nationally or in portions of a country.

¶ Vaccine-associated paralytic polio is a rare (1 in 2.7 million administered doses of OPV) occurrence of paralysis that usually occurs with the first OPV dose; no risk for spreading to others exists. Additional information is available at http://www.who.int/immunization/diseases/poliomyelitis/endgame_objective2/oral_polio_vaccine/VAPPandcVDPVFactSheet-Feb2015.pdf.
